# Psychological factors of college students’ learning pressure under the online education mode during the epidemic

**DOI:** 10.3389/fpsyg.2022.967578

**Published:** 2022-07-28

**Authors:** Leiming Fu, Junlong Li, Yifei Chen

**Affiliations:** ^1^College of Information Management, Nanjing Agricultural University, Nanjing, Jiangsu, China; ^2^College of Public Administration, Nanjing Agricultural University, Nanjing, Jiangsu, China; ^3^Pukou Campus Management Committee, Nanjing Agricultural University, Nanjing, Jiangsu, China; ^4^Faculty of Music, Bangkok Thonburi University, Bangkok, Thailand

**Keywords:** online education, college student study, psychological stress, deep learning, learning pressure

## Abstract

The emergence of the network environment is the product of the combination of the development of computer technology and the development of network technology. Internet technology is slowly penetrating into all aspects of people’s lives and has had a huge impact and change on people’s lives. With the repeated outbreak of the epidemic in recent years, online education has been increasingly applied to the study and life of college students. The epidemic has lasted for 3 years, while the life of college students is only 4 years. In recent years, most of the campus study and life of college students have been carried out in the online education mode. This not only changed the mode of class, but also changed the mental health of college students. Taking the online education model during the epidemic as the research background, this paper selects the psychological factors of college students’ learning pressure to analyze, combined with the design and implementation of the questionnaire, to understand the impact of online education on college students’ cognition, emotion, willpower, and social interaction. The purpose is to find out the psychological factors of college students’ learning pressure under the online education mode, and to propose effective solutions. The analysis of the psychological factors of college students’ learning pressure in the form of questionnaires is more accurate than other forms of experimental investigation, the efficiency is increased by 32%, and the accuracy is also increased by 18%.

## Introduction

The Internet has entered the life of the people. Under the background of the Internet, all aspects of people have been expanded and improved. The online environment has truly entered people’s lives. In this virtual environment, people’s language, thoughts, and visuals are amplified and enhanced by online technology, which can have a devastating impact on their mental health as well as their real lives as they interact with the real environment. The impact of this online education model on students’ mental health differs from traditional education models in that it requires the inclusion of the Internet as an important element in the design and implementation of education, and also requires that the goal of education should not only be to provide traditional mental health education, but also to guide mental health, prevent mental disorders, and regulate online ethics. That is to say, the network has advantages and disadvantages. If you use it well, it will promote the development of life. If you do not use it well, it will lead to earth-shaking changes in life. In recent years, education for college students has mostly been provided in the form of online education, which has not been sufficiently analyzed and researched in terms of psychological changes, psychological health, and intrinsic factors affecting the psychology of students in the online environment (Ospina García et al., 2021). Therefore, it is necessary to investigate the current situation of students’ psychological health using online learning, which is becoming popular, as a research background.

Online education is a way of learning that relies on the Internet. The neoliberal reforms of universities have had a huge impact on higher education and are expected to see more changes in the future. Many of these changes have negatively impacted academic careers, values, and educational experiences (Jain et al., 2022). Feenberg (2017) outlined the main claims and consequences of this rhetorical strategy, and its practical impact on universities to date. The education-focused online platform facilitates active academic discussions being used and reinforces concepts to improve overall course outcomes. Teachers post learning objectives to acknowledge student responses with correct answers and to guide follow-up discussions. In a post-activity qualitative survey, Kolluru S found that most students prefer less stressful online interactions with their peers and teachers (Kolluru and Varughese, 2017). The purpose of Steele J R’s research for the article was to compare the effects of digital interactive educational platforms and standard paper-based education on patients’ knowledge of ionizing radiation (Steele et al., 2017). The purpose of Asif M M for the article study was to observe the impact of the COVID-19 lockdown on the health-related quality of life of undergraduates from online courses at different private institutions (Asif and Javed, 2021). Although research on online education is necessary, it has not been explained by considering the psychological pressure of contemporary college students, so these studies are not comprehensive enough.

Stress is a state of mind that arises in response to something you perceive as stressful. Potthoff S showed that psychological distress is common in mental health services. To date, there is no universally accepted definition of psychological distress (Potthoff et al., 2022). Galvin J used qualitative research methods to conduct one-to-one interviews with 15 clinical psychology trainees. It is important to explore the causes of anxiety and coping strategies to help trainees deal with anxiety more effectively. The findings of this study are discussed in the context of clinical psychology training (Galvin and Smith, 2017). The fast pace of modern online English language teaching and the complexity of teaching relationships make English teachers more stressed in their teaching tasks and more prone to various psychological problems. In addition, Ali I developed a mental health knowledge base and assessment model based on theoretical results and expert experience in the field of English language teaching and psychological stress using fuzzy processing system and fuzzy weighted logical reasoning theory (Ali et al., 2020). With the rapid development of modern football, the professional requirements for referees are also gradually increasing. In this regard, the training of football referees is the most important issue in the university. In the training of football referees, the psychological pressure of referees cannot be underestimated in all aspects. Therefore, Xu Z initially discussed the reasons for the psychological pressure of football referees (Xu, 2018). Psychological stress is an issue that needs attention in contemporary society, but it still needs to be explained in combination with online education to make the article more comprehensive.

The novelties of this paper are as follows: First, through a questionnaire survey, the impact of online education mode on contemporary college students’ psychological pressure during the epidemic has been re-analyzed, making the research more systematic; the impact research mechanism is more clear; the third is the psychological confusion brought to college students by the Internet, which is explored and solved from the perspective of schools, families, individuals, and society.

## Deep learning algorithms

### Deep learning

Deep learning is an algorithm based on artificial neural network and representation learning. It is a core algorithm in machine learning algorithms, and its learning is divided into three types, namely unsupervised, semi-supervised, or supervised (Coates et al., 2017).

#### Backpropagation

Backpropagation is a method of computing the gradient of a function, usually in the form of a neural network. Since there are several hidden layers in the neural network, but there are some small errors in the neurons of each layer, the back-propagation method is required to calculate the errors and optimize them layer by layer.


(1) $$Z^{\left(t\right)}=\left\{ \begin{array}{l} m^{\left(t\right)}-y\\ \Theta^{\left(t\right)}n^{\left(t+1\right)T}.*P^{\prime }\left(a^{\left(t\right)}\right) \end{array}\right.$$

#### Stochastic gradient descent

Gradient descent is like a river flowing from the top of the mountain, the river will flow from the top of the mountain to the foot of the mountain. When the result is to be minimized, it is also called the loss function (Kwak and Lim, 2021). The following assumes a loss function as:


(2) $$M\left(\theta \right)=\frac{1}{2}\overset{a}{\underset{i=1}{\sum }}{\left(J_{\theta }\left(x\right)-y\right)}^{2}$$

**Figure 1:**
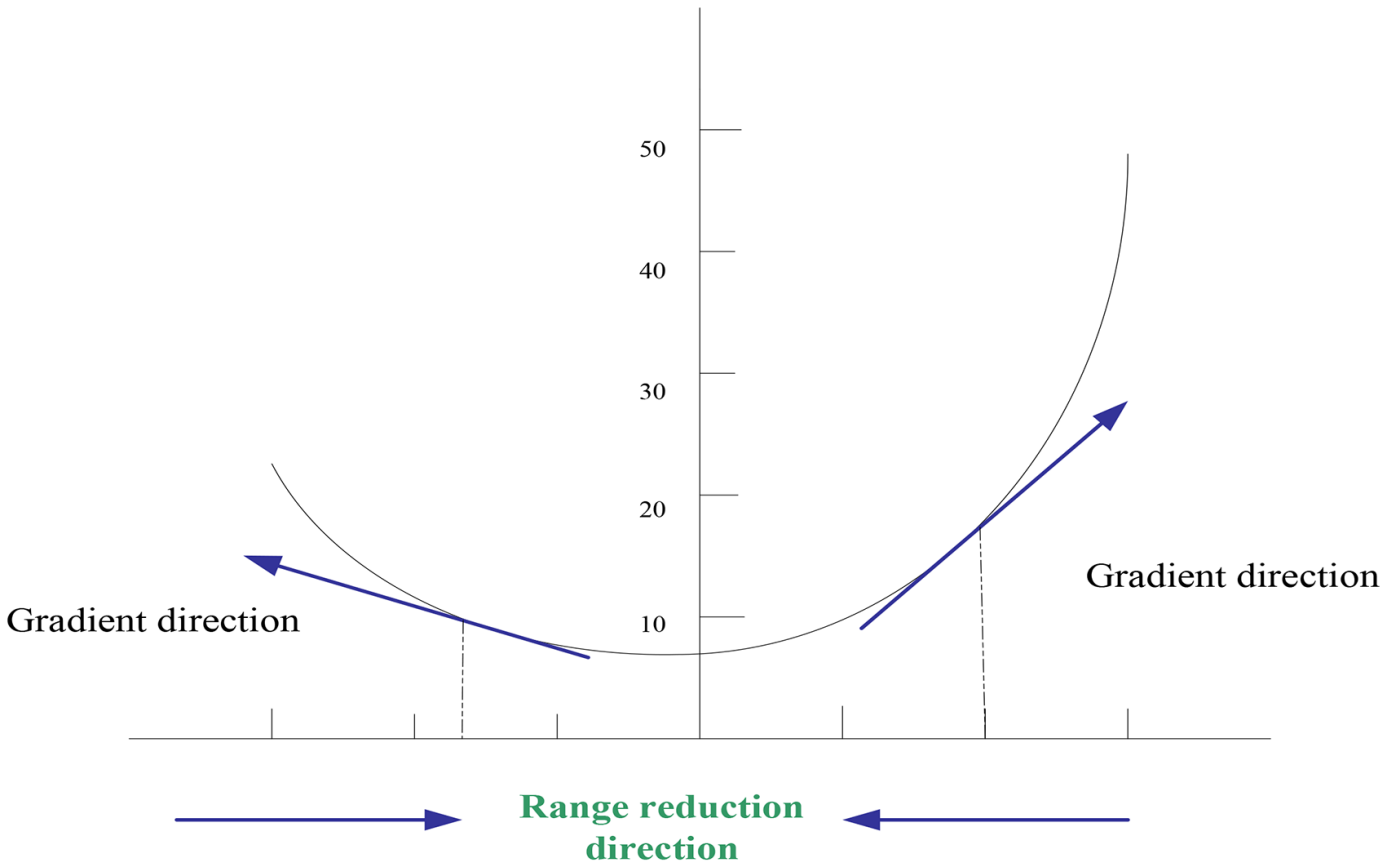
Gradient descent method.

Among them, $J_{\theta }\left(x\right)=\theta_{0}+\theta_{1}x_{1}+\theta_{2}x_{2}\cdots +\theta_{n}x_{n}$ makes it minimized, as shown in Figure 1.

#### Dropout

**Figure 2:**
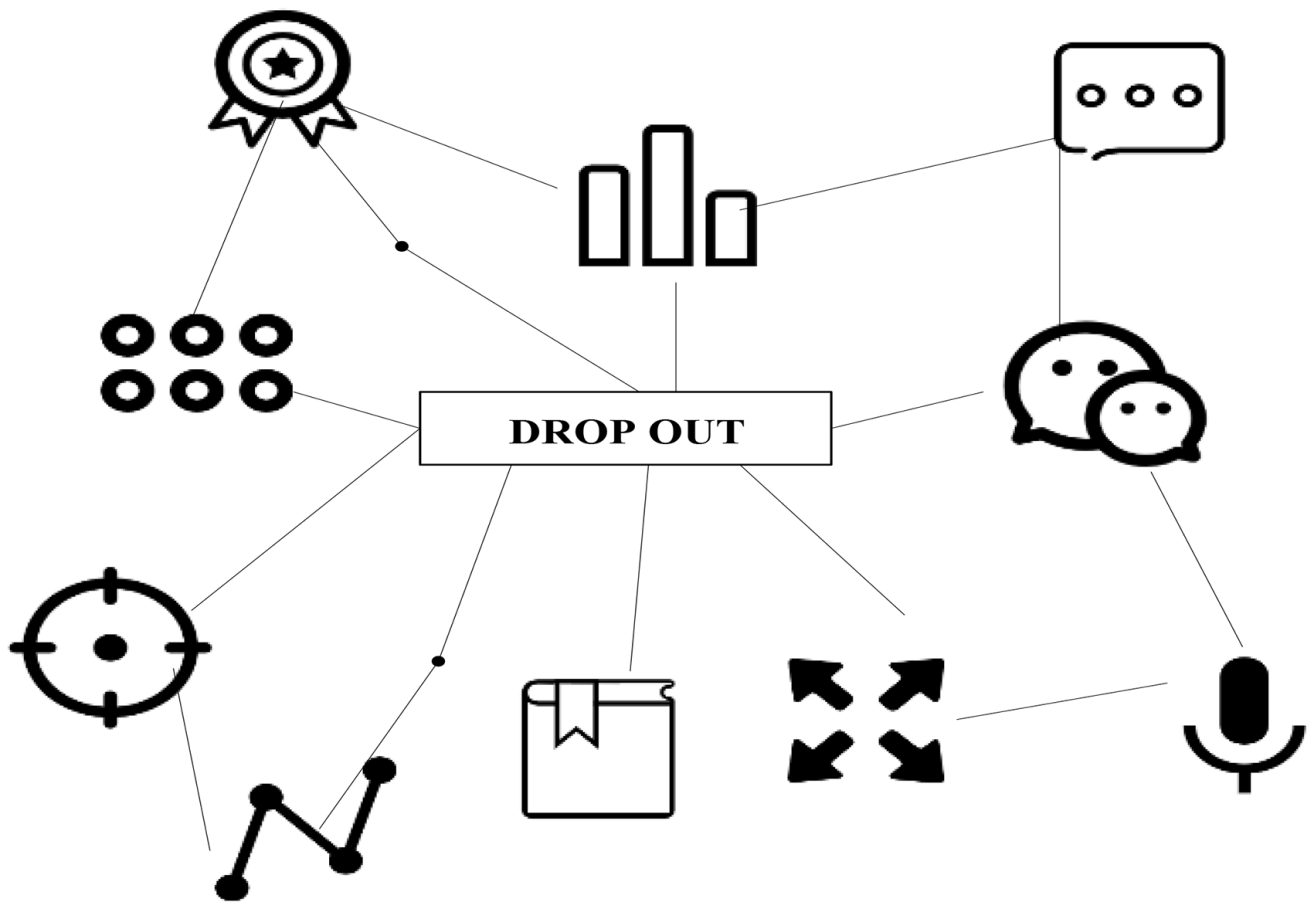
Dropout performance graph.

Dropout is a method to solve the overfitting of neural networks, which is convenient and efficient, so many deep learning users will add dropout to the network to improve performance, as shown in Figure 2.

In the classification task, the simplest voting method to define a probability distribution P(y|k,γ) from the network is used, and the final probability distribution is predicted as:


(3) $$\underset{n}{\sum }P\left(\gamma \right)P\left(\mathrm{y|k,}\gamma \right)$$

γ represents the network, and P(γ) is the probability of this network, which can be defined as a nonstandardized probability distribution:


(4) $${\tilde{P}}_{\mathrm{ensemble}}\left(\mathrm{y|k}\right)=\sqrt[{2^{m}}]{\underset{\gamma }{\prod }p\left(y|k,\gamma \right)}$$

m represents the number of dropped units, and γ represents a parameter in the probability distribution before renormalizing the ensemble:


(5) $$P_{ensemble}\left(y|k\right)=\frac{{\tilde{P}}_{ensemble}\left(y|k\right)}{\sum_{y'}{\tilde{P}}_{ensemble}\left(y|k\right)}$$

##### Long short-term memory network (LSTM)

A recurrent neural network (RNN) is a recurrent neural network that takes sequence data as input and performs recursion in the evolution direction of the sequence connected by all nodes. Long short-term memory network is a variant of RNN. RNN can only have short-term memory because of the problem of gradient disappearance. The LSTM network combines short-term memory with long-term memory using subtle control principles, and overcomes the problem of vanishing gradients to a certain extent (Fisher et al., 2021).

Input:

First, it will take the output $h^{\left(k-1\right)}$ of the previous stage and the input $x^{k}$ of this stage, Because the sigmoid function is often used as the activation function of the neural network, the variables are mapped between 0 and 1, and use sigmoid to control how much to add to the main plot $B_{k}$ , which is the meaning of the first formula. Then, an alternate ${\tilde{B}}_{k}$ is created to use tanh to control how much of the $B_{k}$ is added. Then, by multiplying the two parts together, the total determines how much to affect $B_{k}$ . Adding the effect of the previous forget gate, it can be written as:


(6) $$l^{\left(k\right)}=\alpha \left(W_{l}h^{\left(k-1\right)}+V_{l}x^{\left(k\right)}+a_{l}\right)$$

(7) $$m^{\left(k\right)}=kmnh\left(W_{m}h^{\left(k-1\right)}+V_{m}x^{\left(k\right)}+a_{m}\right)$$

Output:

Hidden state $h^{\left(k\right)}$ is composed of $n^{\left(k\right)}$ and hidden state $B^{\left(k\right)}$ and the tanh activation function, namely,


(8) $$n^{\left(k\right)}=\alpha \left(W_{n}h^{\left(k-1\right)}+V_{n}x^{\left(k\right)}+a_{n}\right)$$

(9) $$h^{\left(k\right)}=n^{\left(k\right)}⊗\tanh\left(B^{\left(k\right)}\right)$$

### Neural networks

**Figure 3:**
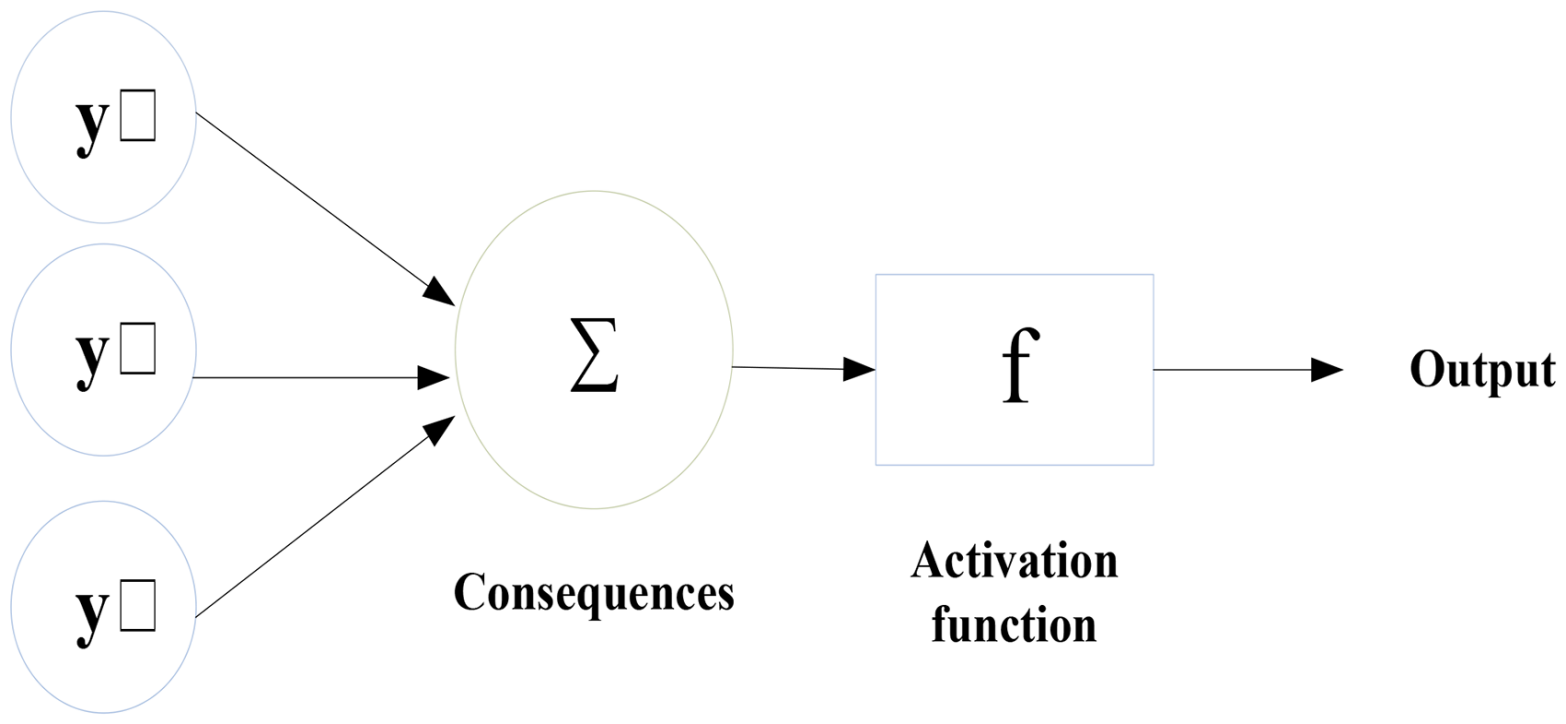
Basic neuron model diagram.

The artificial neural network is an abstraction of the human brain neuron network. By constructing a simple model, different networks are formed according to different connection methods (Khan et al., 2021). The learning process of the artificial neural network is the process of adjusting the input weight and threshold by comparing the difference between the actual output and the expected output. When the error reaches the predetermined requirement, the input–output mapping relationship is established, and the purpose of learning is also achieved. Artificial neurons are the key to artificial neural networks. It is composed of three elements: connection weight, summation unit, and activation function. Each neuron receives information from several other neurons, multiplies them by their assigned weights, adds them together, and gives the sum to the following neuron, as shown in Figure 3.

#### Forward neural network

**Figure 4:**
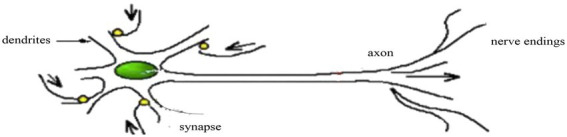
Forward neural network model diagram.

The forward neural network connects the neurons of the front and rear layers together, as shown in Figure 4.

The forward algorithm is a process from the input layer to the hidden layer to the output layer. Add the value of the node $m_{1}$ to be calculated, it can be obtained as follows:


(10) $$net_{m_{1}}=a_{1}\times n_{1}+a_{2}\times n_{2}+b_{1}\times 1$$

This is a simple weighted summation, and when calculating node $q_{1}$ , the output of node $m_{1}$ cannot simply use the result of $net_{m_{1}}$ , and the activation function must be calculated, that is, the “features of activated neurons” are retained and mapped by the function come out. Taking the sigmoid number as an example, the output of $m_{1}$ :


(11) $$out_{m_{1}}=\frac{1}{1+e^{-net_{m_{1}}}}$$

Then,


(12) $$net_{q_{1}}=out_{m_{1}}\times n_{5}+out_{m_{2}}\times n_{6}+b_{2}\times 1$$

In the actual situation, it depends on the randomly given weights, so the error between the result obtained with a high probability and the actual result is relatively large. At this time, it is necessary to compare the difference between the output and the actual result, recalculate the difference, and adjust the weight relationship in the network (Benrabah et al., 2020). The main calculation steps are as follows:

First calculate the total error:


(13) $$E_{total}=\sum \frac{1}{2}{\left(t\arg et-output\right)}^{2}$$

Then update the weights of the hidden layer:


(14) $$\frac{\partial E_{total}}{\partial out_{q_{1}}}\times \frac{\partial out_{q_{1}}}{\partial net_{q_{1}}}\times \frac{\partial net_{q_{1}}}{\partial n_{5}}$$

Last updated weights:


(15) $$n_{5}^{+}=n_{5}-\beta \times \frac{\partial E_{total}}{\partial n_{5}}$$

#### Convolutional neural network

Convolutional neural networks only allow some neurons between two layers to be connected, not all neurons between layers are connected.

First-order continuous form:


(16) $$D\left(x\right)=\int_{-\infty }^{+\infty }f\left(t\right)g\left(x-t\right)dt$$

Second-order discrete form:


(17) $$Conu_{i,j}=\overset{A-1}{\underset{a=0}{\sum }}\overset{B-1}{\underset{b=0}{\sum }}w_{a,b}x_{i-a,j-b}$$

For the above formula, it can be simplified to:


(18) $$CrossCorr_{i,j}=\overset{C-1}{\underset{c=0}{\sum }}\overset{D-1}{\underset{d=0}{\sum }}w_{c,d}x_{i+c,j+d}$$

#### Recurrent neural network

For any sequence time k, hidden state $h^{\left(k\right)}$ is obtained from $x^{\left(k\right)}$ and $m^{\left(k-1\right)}$ :


(19) $$m^{\left(k\right)}=\alpha \left(s^{\left(k\right)}\right)=\alpha \left(Vx^{\left(k\right)}+Wm^{\left(k\right)}+a\right)$$

α is the activation function, and the sequence time k, the output formula of the model is relatively simple:


(20) $$P^{\left(k\right)}=Uh^{\left(k\right)}+b$$

Finally, at the sequence time k, the predicted output is:


(21) $${\hat{y}}^{\left(k\right)}=\alpha \left(p^{\left(k\right)}\right)$$

## Influencing factors of psychological stress under the online education model

### Experimental method

The first-hand information about the writing of the thesis was obtained through the questionnaire survey, and then the research and collaboration of the article were carried out based on the results of the questionnaire survey.

### Experimental standard

#### Correct concept and consciousness

The key to judging a person’s mental health is whether he can implement cognition that conforms to objective facts under normal intellectual conditions. It can correctly identify a large amount of information in the network and properly handle the relationship between the network and reality. Good self-control, able to identify and resist various mental health disorders. There are normative ethical constraints in the process of using the Internet.

#### Maintain the unity of personality in the two environments of network and reality

The virtuality, imagination, and diversity of the Internet allow college students to interact and express their opinions in the Internet in any role at will, which is obviously different from the real environment, and also causes the dual personality of college students (Buhren and Trger, 2022). This kind of personalities in the network environment and the real environment may sometimes be harmonious and unified, and sometimes, they may be seriously antagonistic, resulting in serious mental health disorders, which requires college students to maintain the unity of personality between the network and reality.

#### Both the network and the real environment have good emotions

Good emotions can make college students feel happy, optimistic, and calm, and then have a strong sense of social responsibility, so that they can study and live better. However, due to the lack of moral supervision, the Internet can easily become a tool for college students to vent their negative emotions. Over time, the Internet loses its original emotion regulation function, which increases the negative emotions of college students and causes mental health disorders (Brooks et al., 2020).

#### Sound will

It means that college students can correctly identify the relationship between the Internet and reality, and can resist all kinds of tempting unhealthy information in the Internet. It is necessary to reasonably control the time spent on the Internet and not be addicted to the Internet; after being frustrated in real life, you can choose the correct stress relief method, rather than simply relying on the Internet to vent your emotions and stress.

#### Maintain good interpersonal relationships

This includes the skills of college students to maintain good interpersonal relationships with others in a virtual network environment, as well as to maintain good interpersonal relationships with others in a real environment, without the influence of the Internet causing a decline in interpersonal skills.

#### Leaving the network environment will not cause physical and mental discomfort

Under the circumstance that college students’ willpower declines and their identification ability is insufficient, long-term use of the Internet will lead to an obvious sense of dependence on the Internet. If the network environment is leaved, it will cause physical and mental discomfort such as anxiety, loneliness, irritability, and discomfort, which is also a manifestation of psychological unhealthy (Lei, 2019).

### Experiment implementation

#### Questionnaire survey design and data processing

The content of the questionnaire covers whether the online education mode has any impact on college students’ cognition, emotion, interpersonal communication, and will. And the influencing factors also cover the three aspects of family, society, and school, which ensures the comprehensiveness of the survey results.

This paper uses Excel to conduct data processing and analysis on the results of the questionnaire survey. The analysis process mainly follows the following steps.

#### Research objects

The samples are from 2019, 2020, 2021, and 2022, and the number of samples in each grade is basically the same. The specific distribution of the samples is shown in Table 1.

**Table 1:** Survey respondents’ profile.

Gender	Man	68	42.5%
	Woman	92	57.5%
Major	Liberal arts major	84	52.5%
	Science major	76	47.5%

### Survey results

#### Analysis of impact results

1. The influence of online education mode on college students’ learning cognition.

**Figure 5:**
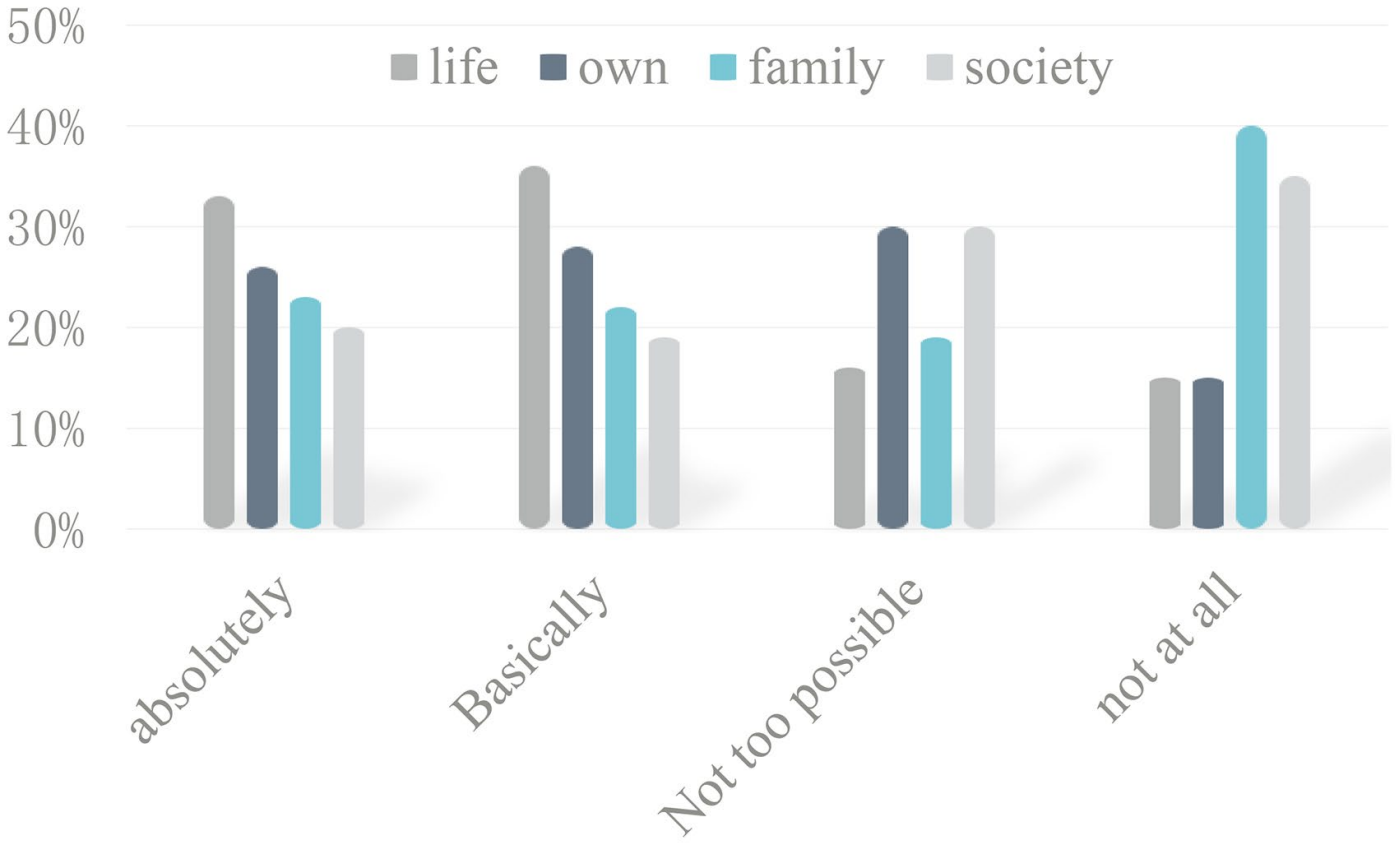
The impact of online education mode on college students’ learning cognition.

As shown in Figure 5, those who think they will be completely subject to change are by 33%, while 36% believe that it will basically change, 16 and 15% think they will not change at all and they will not change at all.

At the same time, 23 and 22% of college students believe that the online education model can change the views of college students on family. While 40% thought it was unlikely to change, 35% of college students would not be affected when online education models change how college students view society, indicating that although the network environment will change college students’ cognition and view of themselves, life, and society, it still cannot play a fundamental role in changing family cognition.

2. The influence of online education mode on college students’ personality.

**Figure 6:**
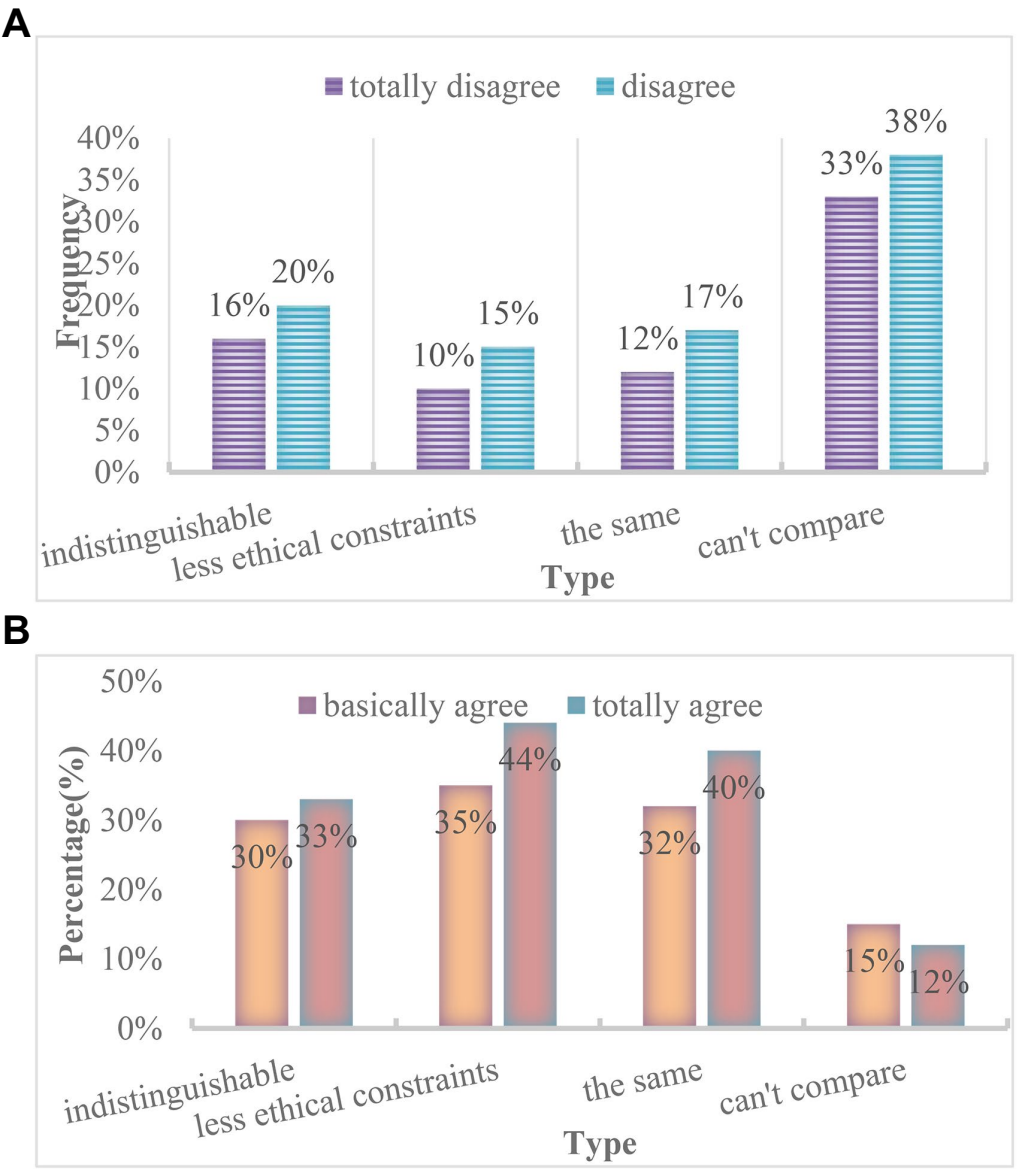
The influence of online education mode on college students’ personality. **(A)** Freshmen. **(B)** Senior students.

In the college life accompanied by the epidemic, whether the network and reality maintain a consistent personality is one of the criteria for evaluating mental health. Compared with the traditional education model, the online education model lacks the supervision of teachers, and the college students who lack autonomy will stay in the online life for a long time. While the Internet brings various conveniences to college students, it also brings a lot of unhealthy information. Being in such an environment for a long time will inevitably lead to unsound personality development of college students with weak judgment (Brosch and Binnewies, 2018). For example, although online games can bring entertainment and entertainment to college students, long-term addiction to the Internet will not only cause autism, but also Internet addiction. Under such a background, it is not difficult to understand that in the network environment, college students will be self-enclosed and unwilling to communicate with others face-to-face, which will seriously lead to the split of college students’ personality. Here, the statistical results of freshman and senior students are analyzed, as shown in Figure 6.

3. The influence of online education mode on college students’ emotions.

During the epidemic, many students did not study seriously and spent most of their time online. Due to the lack of communication with teachers and classmates and the absence of moral and behavioral constraints from society, school, and family, they are more likely to express their true feelings and vent their resentment toward others and society in the online environment. At the same time, students can also create their own websites and spaces according to their own ideas and interests, forming an alter ego in the online environment (Li, 2019). Emotion is also a measure of college students’ mental health, as shown in Table 2.

**Table 2:** The influence of college students’ emotions under the online education model.

Manifestations	Number	Frequency (%)
Less communication with teachers	97	48.5
Less communication with classmates	36	18
Communicate more with parents	55	27.5
No difference	12	6

**Figure 7:**
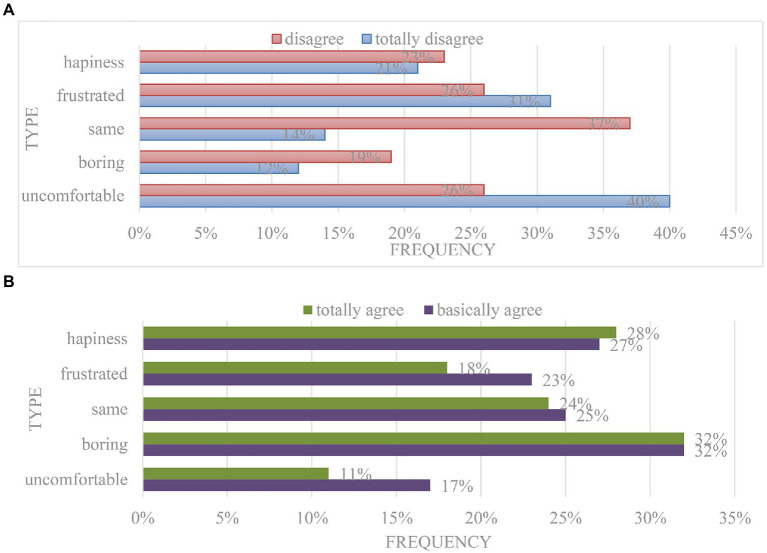
The influence of online education mode on college students’ mood. **(A)** Freshmen. **(B)** Senior students.

First of all, when college students receive and process information in the network environment, it will lead to their emotional ups and downs, resulting in low interest in learning, irritability, and inability to study with peace of mind, resulting in emotional damage and inability to study normally. Secondly, in the relaxed and free network environment, college students are no longer subject to various constraints of the real environment, thus losing initiative and consciousness (Zarin et al., 2019). Thirdly, the network environment will make college students alienate the interpersonal relationships in the real environment. They will take advantage of the lack of moral supervision in the network environment to vent their emotions, destroy the trust between people, and cause damage to their own emotions. Finally, the network environment lacks the interaction between people, and cannot obtain the experience of true emotional integration, which makes their emotions become more and more indifferent, as shown in Figure 7.

4. The influence of online education mode on the will of college students.

Personal will plays an important role in mental health, and people with strong will can easily resist the influence of the external environment on their psychology. In terms of online education, the will is reflected in whether college students can earnestly study by themselves. If it can be made full use of the network for learning or necessary communication, it means that it has a good level of mental health; otherwise, it means that its mental health level is low.

Online education is carried out with the help of computers or mobile phones. The convenience and entertainment of the Internet will attract college students, especially in large classes. Boring classes are easy to distract, and at this time, the Internet can provide a space for college students to relieve stress, so that they can abandon the original class, resulting in no learning at all (Yu et al., 2021).

**Figure 8:**
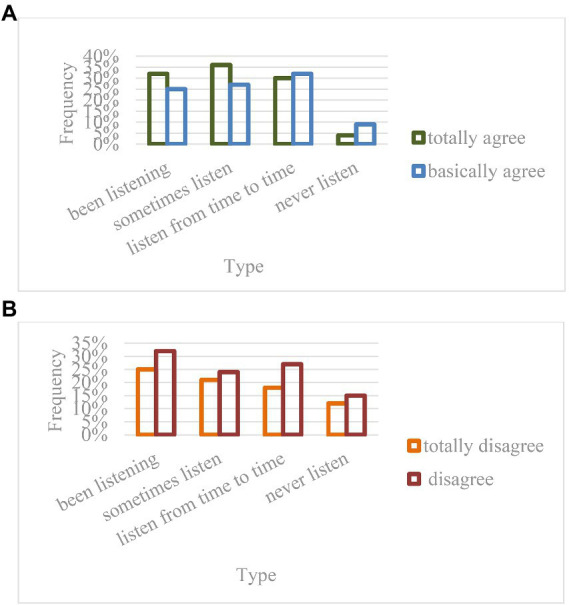
The influence of online education mode on college students’ will. **(A)** Freshmen. **(B)** Senior students.

At present, college students are facing very severe academic, employment, and emotional pressure, and online education has less constraints from schools. When college students feel wronged and dissatisfied in the real environment, they will rely on the network environment to achieve psychological balance, which will weaken their ability to withstand setbacks over time, as shown in Figure 8.

5. The influence of online education mode on college students’ social interaction.

**Figure 9:**
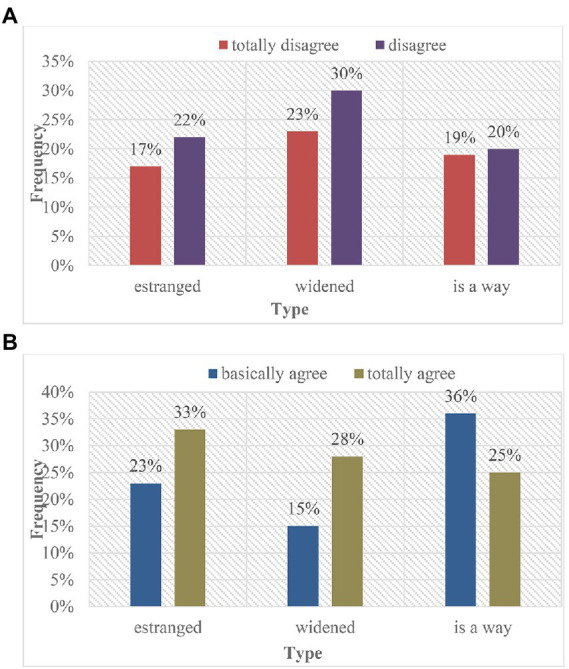
The impact of online education mode on college students’ social interaction. **(A)** Freshmen. **(B)** Senior students.

The long-term online education model during the epidemic has given many college students no opportunity to expand their circle of friends, and some even closed themselves (Choi et al., 2021), as shown in Figure 9.

Although 25% of college students believe that family members and relatives are their most frequent social circle, 44% of college students think their classmates are their most common social circle, 15% of college students think their former friends are their most common social circle, but it cannot be ignored that 16% of college students still think that their friends on the Internet are their most frequent social circles, as shown in Table 3.

**Table 3:** The most common social circles of college students in the online education model.

Social crowd	Number	Frequency (%)
Family	50	25
Classmates	88	44
Friends	30	15
Netizens	32	16

#### Analysis of the causes of influence

Family education concept and family growth environment have far-reaching influence on college students, and many family concepts even affect children’s life. The Chinese-style family education concept deeply influenced by Chinese traditional culture has its own advantages and its inevitable shortcomings.

Families are educating their children and society is educating young people. When mistakes are made, they are often told that there is an opportunity to correct them. In particular, the tolerance of young people sometimes becomes a kind of tolerance, and this tolerance is based on others including themselves (Kim et al., 2016). During the epidemic period, college students under the online education model have been at home for a long time, and some parents simply do not care whether their children are in class well, resulting in even worse class results. Students who have been at home for a long time will have a more harmonious relationship with their parents, but most students have a worse relationship with their family. Because Chinese parents still prefer repressive education methods, they will want to criticize education when they see their children at home every day, which will lead to greater psychological pressure on college students.

In addition to the family, the most important influence on a student is the campus living environment. In the past, there were teachers and counselors in the school life, but now during the epidemic, all education is online, which has caused college students to have more free time all of a sudden. Almost all the students in the dormitory are holding computers, playing games, or watching bubble dramas. Internet access has become their biggest hobby to pass their free time, and they spend more time on their mobile phones instead of enriching themselves (El-Rashidy et al., 2022).

Especially in contemporary society, it is an indisputable fact that people’s beliefs are lacking. The less firm beliefs are, the easier it is to be penetrated by bad information on the Internet. Especially in the online world, people can hide their true identities, even without regard to the bottom line of morality.

#### Countermeasures

Regarding the path of college students’ psychological education under the online education mode during the epidemic, the path is mainly based on the Internet, and the method of college students’ psychological health education is innovated through the Internet. There are many forms of online psychological education activities, such as online psychological classrooms, psychological health public platforms, class blogs, psychological health QQ groups, psychological health education software, and so on. The development of these activities is to make full use of the Internet, a convenient and familiar way for college students, to transmit mental health knowledge, content, methods, and ideas. Through the development of various online psychological education activities, college students can not only fully feel the convenience brought by the progress of network technology, but also establish a correct mental health concept and guide their psychological healthy development through the development of activities (Chau et al., 2021).

Colleges and universities can disclose the emails of psychological education and psychological counseling workers to college students, and inform students that they can contact their psychological problems and confusions by email and seek solutions. Psychological educators who receive emails should answer the questions raised by students in a timely manner, so as to help the seekers form a sound self-psychological concept and the ability to adapt to the outside world.

This is an instant information exchange method, which is significantly different from the first two types of methods. It requires experts or psychoeducational staff to be on duty at a certain time period. Chat can be conducted through QQ, WeChat, or in a dedicated chat room. The chat methods can be divided into text chat and voice chat (Xueyan, 2017). Due to the immediacy of information transmission, the effect of text chat is far stronger than that of traditional letter consultation, and voice consultation has a better intervention effect on some college students’ psychological crisis.

The biggest feature of the web conference is that it can use camera technology to allow experts, workers, and college students to have face-to-face conversations. The most intuitive visual perception of college students’ voice, intonation, facial expressions, body movements, etc., can be used to judge their mental health status through network technology. At the same time, the online conference also breaks the time and geographical constraints, making the exchange and interaction of information possible (Kosif and Cinpolat, 2017). Although this is a relatively expensive method now, with the advancement of technology, it will benefit many college students’ mental health education.

Families are the harbor for students’ spiritual home. Although college students are basically independent, their psychological development is still relatively immature and needs the care of their parents. Parents should pay more attention to the mental and healthy growth of students, and should give high attention and cooperation in cultivating the physical and mental health of college students.

## Conclusion

Since the network technology entered people’s life, the network has changed the way people live, study, and work. It can be known that a large number of college students studied in the online education mode during the epidemic. This can be good or bad, those with high self-control will study hard, and those with low self-control may be depressed and addicted to the Internet. Due to the characteristics of virtuality, complexity, and openness of the online world, coupled with the mistakes of college students’ cognition of the Internet, long-term addiction to the Internet brings emotional loss and weakening of interpersonal relationships. The analysis of the psychological factors of college students’ learning pressure in the form of questionnaires is more accurate than other forms of experimental investigation, the efficiency is increased by 32%, and the accuracy is also increased by 18%. There are still some deficiencies in the research of this paper. The discussion of psychological stress in the paper is not comprehensive enough. It will be continuously strengthened and improved in future research.

## Data availability statement

The original contributions presented in the study are included in the article/supplementary material, further inquiries can be directed to the corresponding author.

## Author contributions

LF: Writing—original draft preparation. JL and YC: Editing data curation and supervision. All authors contributed to the article and approved the submitted version.

## Funding

This work was supported by the Education Reform Fund of Nanjing Agricultural University. Exploration and reflection on virtual experiment teaching of science and engineering based on the combination of “Internet plus” online and offline (ID: 2021Y074). This work was also supported by the Ministry of Education of the People’s Republic of China Industry-University Cooperation Collaborative Education Project. Research on the reform of higher education personnel training mode under the background of Industry 4.0 (ID: 202102218003).

## Conflict of interest

The authors declare that the research was conducted in the absence of any commercial or financial relationships that could be construed as a potential conflict of interest.

## Publisher’s note

All claims expressed in this article are solely those of the authors and do not necessarily represent those of their affiliated organizations, or those of the publisher, the editors and the reviewers. Any product that may be evaluated in this article, or claim that may be made by its manufacturer, is not guaranteed or endorsed by the publisher.
